# Anti-toxine

**Published:** 1897-09

**Authors:** 


### ANTI-TOXINE.

"It has been said that I have been prejudiced against the use of anti-toxine. If there is a specific against diphtheria I want it. No member of this Academy, no member of the profession in any portion of the world, needs it more than I do. *I could not possibly have brought myself to the decision in which I find myself to-night had it not been for my strong conviction regarding the injurious effects of anti-toxine. It is because I believe that it is dangerous* that my convictions compel me to speak. The time will come, gentlemen, *when every member of this Academy will feel with relation to it as I do to-night*, and you will come to my conviction, as various members have already done."—*Our Dumb Animals.*

